# Transcatheter Mitral Valve Repair in Surgical High-Risk Patients: Gender-Specific Acute and Long-Term Outcomes

**DOI:** 10.1155/2016/3934842

**Published:** 2016-03-02

**Authors:** Eike Tigges, Daniel Kalbacher, Christina Thomas, Sebastian Appelbaum, Florian Deuschl, Niklas Schofer, Michael Schlüter, Lenard Conradi, Johannes Schirmer, Hendrik Treede, Hermann Reichenspurner, Stefan Blankenberg, Ulrich Schäfer, Edith Lubos

**Affiliations:** ^1^Department of General and Interventional Cardiology, University Heart Center, University Medical Center Hamburg-Eppendorf, Martinistrasse 52, 20246 Hamburg, Germany; ^2^Department of Cardiology, Asklepios Klinik St. Georg, Lohmühlenstraße 5, 20099 Hamburg, Germany; ^3^Department of Cardiovascular Surgery, University Heart Center, University Medical Center Hamburg-Eppendorf, Martinistrasse 52, 20246 Hamburg, Germany

## Abstract

*Background*. Analyses emphasizing gender-related differences in acute and long-term outcomes following MitraClip therapy for significant mitral regurgitation (MR) are rare.* Methods*. 592 consecutive patients (75 ± 8.7 years, 362 men, 230 women) underwent clinical and echocardiographic follow-up for a median of 2.13 (0.99–4.02) years.* Results.* Significantly higher prevalence of cardiovascular comorbidities, renal failure, and adverse echocardiographic parameters in men resulted in longer device time (*p* = 0.007) and higher numbers of implanted clips (*p* = 0.0075), with equal procedural success (*p* = 1.0). Rehospitalization for heart failure did not differ (*p*[logrank] = 0.288) while survival was higher in women (*p*[logrank] = 0.0317). Logarithmic increase of NT-proBNP was a common independent predictor of death. Hypercholesterolemia and peripheral artery disease were predictors of death only in men while ischemic and dilative cardiomyopathy (CM) and age were predictors in women. Independent predictors of rehospitalization for heart failure were severely reduced ejection fraction and success in men while both ischemic and dilative CM, logistic EuroSCORE, and MR severity were predictive in women.* Conclusions*. Higher numbers of implanted clips and longer device time are likely related to more comorbidities in men. Procedural success and acute and mid-term clinical outcomes were equal. Superior survival for women in long-term analysis is presumably attributable to a comparatively better preprocedural health.

## 1. Introduction

Severe mitral regurgitation (MR) is characterized by poor prognosis, often impaired by collateral comorbidities [1]. MitraClip therapy has emerged as an established method with high success rates due to growing experience [2, 3]. In this heterogeneous patient population, numerous analyses have been performed to stratify the procedural outcome, mainly focusing on echocardiographic variables as well as preexisting conditions [4–6]. Investigations in transcatheter aortic valve replacement trials have demonstrated gender-related differences suggesting female gender as beneficial regarding short-, mid-, and long-term outcomes [7–9]. In contrast, male gender has previously been reported to have a significantly better long-term survival following mitral valve surgery while female gender emerged as an independent risk factor for mortality following valvular heart surgery [10, 11]. Yet, characteristics of patients treated with surgical or percutaneous approach for MR differ fundamentally [12]. These diverse findings emphasize the necessity to provide distinguished gender-specific analyses of interventional methods. Recently published data demonstrated safety and efficacy of MitraClip therapy in two cohorts of patients treated with MitraClip regardless of gender up to 12 months after clip implantation [13, 14]. However, long-term data of a large cohort is missing to date. Hence, we sought to characterize a collective who underwent MitraClip procedure at our institution to provide a refined analysis on gender-related differences in acute and long-term outcomes.

## 2. Methods

### 2.1. Study Participants

From September 2008 until April 2015, 592 consecutive patients (75 ± 8.7 years) with moderate-to-severe or severe MR underwent MitraClip therapy (Abbott Vascular, Redwood City, California) at our center. All patients (mean logistic EuroSCORE 21.0, mean Society of Thoracic Surgery mortality score 4.3) were adjudicated as not amenable to surgery by heart team consensus prior to the intervention. The study population was divided according to gender (men 61.1%, women 38.9%). All patients provided written informed consent and the study was approved by the local ethics committee.

### 2.2. Clinical Follow-Up

All patients underwent clinical examination and completed a six-minute walk test (6MWD) and the Minnesota Living with Heart Failure Questionnaire (MLHFQ) at baseline, 6 and 12 months after intervention, and annually thereafter. A full history of comorbidities and reasons for rehospitalization was obtained and recorded at each visit. If death occurred, documentation was retrieved from the patient's family practitioner. If a visit at the study site was not possible, data was obtained by structured telephone interview by professional study personnel. New York Heart Association (NYHA) grading defined clinical phenotype. Blood samples were taken before (1–3 days) and after (2–5 days) MitraClip implantation and at each site visit and immediately analyzed at the University Medical Centre's central laboratory for levels of C-reactive protein (CRP), creatinine, gamma glutamyl transferase (yGT), aspartate transaminase (AST), alanine aminotransferase (ALT), N-terminal pro-brain natriuretic peptide (NT-proBNP), and troponin T. Glomerular filtration rate (GFR) was calculated according to the modification of diet in renal disease (MDRD) formula.

### 2.3. Echocardiography

Echocardiographic measurements of the MR at baseline and after intervention, left ventricular (LV) end-systolic and end-diastolic diameters, and volumes were obtained according to the American Society of Echocardiography and European Association of Echocardiography guidelines [15, 16]. Additionally, mitral valve orifice area, regurgitant volume, regurgitant fraction, LV total stroke volume (TSV), and forward stroke volume (FSV) were measured as previously published [4]. Mitral regurgitation severity after intervention was assessed as proposed by Foster et al. [17]. Echocardiographic measurements were performed at each study site visit.

### 2.4. Transcatheter Mitral Valve Repair Procedure

All Mitra-Clip interventions were performed by experienced operators according to previously published protocols [2]. Acute procedural success was defined as placement of 1 or more clips resulting in a residual MR grade of ≤2+, graded by echocardiography. Mid- and long-term success were defined accordingly.

### 2.5. Study Endpoints

Study endpoint for survival analysis was all-cause mortality. Decompensation for heart failure was considered as endpoint for freedom-from-rehospitalization analysis. The median follow-up time for the overall population was 2.13 (0.99–4.02) years.

### 2.6. Statistical Analyses

Continuous variables were presented as mean and standard deviation and 25th and 75th percentile for skewed variables or as median plus interquartile range (IQR) and discrete variables as absolute and relative frequencies per category. Comparisons of continuous variables were performed using Wilcoxon signed rank test for paired samples and Mann-Whitney *U* test for unpaired samples as well as *t*-test. Comparisons of categorical variables were performed by Fisher exact (for 2 × 2 tables) or chi-square test (for [*n* > 2] × 2 tables). Kaplan-Meier curves for survival and freedom from rehospitalization were assessed and log-rank test was performed. Univariate analyses were calculated by cox regression analysis and presented as hazard ration (HR) with 95%-confidence interval (CI). Multivariate analyses for death and rehospitalization for heart failure were performed by stepwise backward analysis (Akaike information criterion parameter *k* = 3) including all parameters of univariate cox regression analysis. Furthermore, multivariate cox regression analysis adjusted to parameters from stepwise backward analysis was performed. Additionally, multivariate cox regression analysis of the study population adjusted to cardiovascular risk factors was performed. The *p* values were two-sided and <0.05 was considered statistically significant. Analyses were performed using* R Software, Version 3.2.1* (R Development Core Team (2009),* R: A Language and Environment for Statistical Computing*, R Foundation for Statistical Computing, Vienna, Austria. ISBN 3-900051-07-0, URL http://www.R-project.org).

## 3. Results

### 3.1. Baseline Characteristics of the Study Population

Table 1 displays the baseline clinical characteristics of the study population divided according to gender. Age, body mass index, and classical risk factors as diabetes mellitus and smoker status were significantly higher in men. Coronary artery disease including previous myocardial infarction, percutaneous intervention, and coronary artery bypass grafting were more often represented in male patients which translated into higher prevalence of ischemic cardiomyopathy with lower LV ejection fraction (39.0 ± 15.3 versus 46.5 ± 15.1, *p* < 0.001) and significantly higher logistic EuroSCORE. The rate of implanted cardiac resynchronization therapy (CRT) devices was significantly higher in male patients. Baseline echocardiographic parameters are presented in Table 4. Higher LV end-diastolic and end-systolic volumes with wider LV diameters were observed in men. Total stroke volume, regurgitant volume, and regurgitant fraction were all significantly higher in men. Additionally, renal failure was significantly overrepresented in male patients. These findings were not represented in clinical phenotypes with higher measures in 6MWD in male patients and no significant differences in NYHA functional classification or MLHFQ score rates, as shown in Table 3.

### 3.2. Intraprocedural Differences

As provided in Table 2, more clips were implanted in men which corresponded to significantly longer device and procedural and radiation times. However, device success (MR grade ≤2+ at discharge) did not differ significantly.

### 3.3. Postprocedural Clinical Outcome

At discharge, NYHA functional class had improved in both genders with 56.4% of the male and 60% of the female patients in NYHA functional classes I and II. These findings remained stable throughout the whole observation period (male 62.9%, female 58.2% in NYHA functional classes I and II at 12 months; male 61.1%, female 53.5% at 24 months). Apart from beneficial results regarding subjective quality of life as obtained by MLHFQ for men at discharge, no difference over time was observed. Male patients showed a greater physical performance at initial presentation, as obtained by 6MWD (Table 3). Furthermore, a greater increase of the walking distance in male patients was determined at discharge and at 12 months. No difference was assessed at 24 months. Higher creatinine levels were observed in male patients throughout the entire observation period. Gamma glutamyl transferase and troponin T were higher in men throughout the study.

### 3.4. Postprocedural Echocardiographic Outcome

Mitral regurgitation severity at discharge was reduced to MR ≤2+ in 91.1% of men and 90.5% of women. This grading remained high at 12 months (91.1% for men, 85.5%) and 24 months (92.1% for men, 80.8% for women) with no significant gender-related differences. Corresponding to these results, there was a decrease in TSV and an increase in FSV with significant gender-related differences at discharge and again for FSV after 24 months, both favoring male patients (Table 4).

Long-term data showed no change in the comparative relation of LV ejection fraction in both genders.

### 3.5. Long-Term Follow-Up

**Figure 1:**
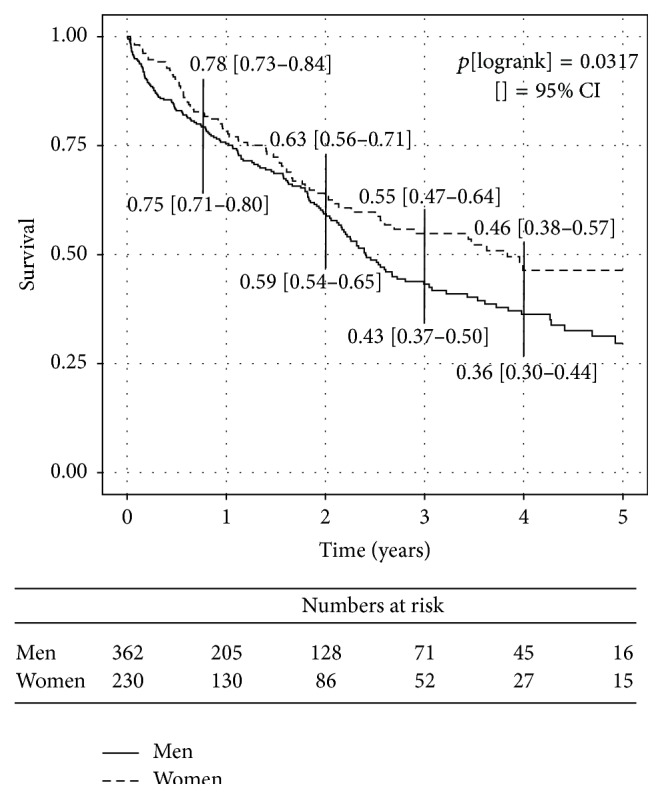
Kaplan-Meier curves presenting the cumulative incidence of death of all causes during follow-up according to gender.

**Figure 2:**
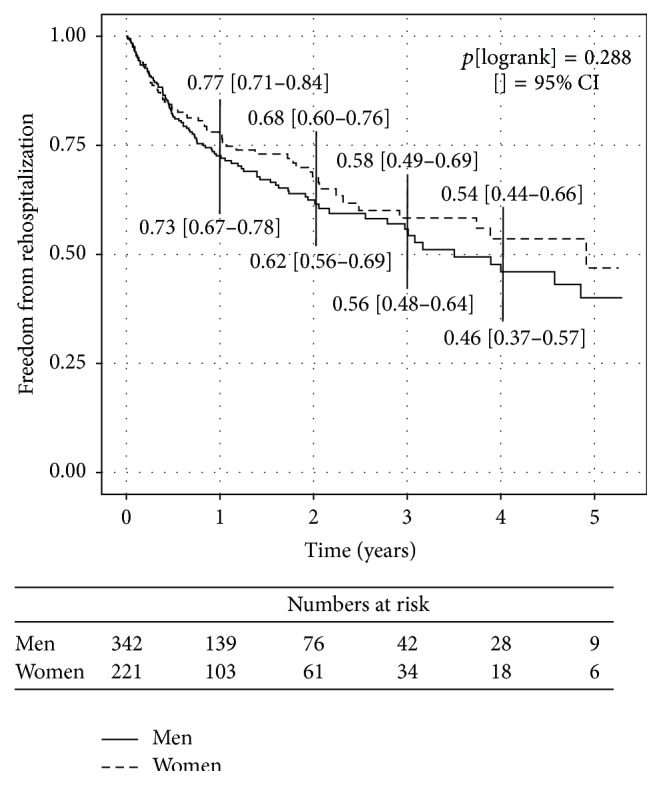
Kaplan-Meier curves presenting the incidence of rehospitalization for heart failure during follow-up according to gender.

Kaplan-Meier estimates for freedom from death showed a significant better survival in women (Figure 1), whereas Kaplan-Meier estimates for freedom from rehospitalization for heart failure (Figure 2) did not meet significance. The combined endpoint of both was barely significant (*p*[logrank] = 0.0492; data not shown) displaying advantages for female gender.

### 3.6. Predictors of Death and Rehospitalization

Univariate cox regression analysis revealed renal failure and NT-proBNP as predictors of death in both genders (Table 5). Hypercholesterolemia, coronary artery disease, success rate, MR severity 4 versus 3, age, and body mass index were univariate predictors of death solely in men. In women, diabetes and nicotine abuse reached significance as univariate predictors of death. Logistic EuroSCORE was significant as predictor of death and rehospitalization for heart failure in women (Table 6). Hypertension, LV ejection fraction <30%, success rate, and logarithmic increase of NT-proBNP were predictors of rehospitalization for heart failure in men, yet not in women.

Stepwise backward regression analysis using all variables from the univariate cox regression analysis revealed diabetes, hypercholesterolemia, atrial fibrillation, NT-proBNP, and peripheral artery disease as independent predictors of death in men (Table 7, Panel A). Except diabetes and atrial fibrillation, all variables remained independent after adjustment for all significant predictors (Table 7, Panel B). Age, both ischemic and dilative cardiomyopathy, and logarithmic increase of NT-proBNP were significant predictors of death in women after stepwise backward analysis and remained so after multivariate adjustment (Table 8, Panels A and B). Regarding predictors of rehospitalization for heart failure, LV ejection fraction <30% was an independent predictor in men. Success rate was shown to be protective in men. In women, logistic EuroSCORE and ischemic and dilative cardiomyopathies were significant even after multivariate analysis, as was MR severity though not being significant in stepwise backward regression analysis.

Male gender was not a predictor of either death or rehospitalization for heart failure in multivariate cox regression analysis (Table 9).

## 4. Discussion

Results of our large scale, real world analysis of 592 patients treated with MitraClip suggest an effective treatment of MR disregarding gender with a higher mortality in men in long-term analysis. Despite adverse baseline parameters there was no gender-specific difference in acute and mid-term outcomes.

Gender-related outcome analyses following TMVR are scarce. Yet, data on patients undergoing mitral valve surgery have previously shown a higher perioperative mortality and poorer long-term survival in female patients [18] and the STS risk model emphasizes female sex as risk factor for mortality with a hazard ratio of 1.39 [19]. However, there are major differences, which limit the comparability of data following surgical versus transcatheter approaches. First, Vassileva et al. showed that the mitral valve repair rate in women was significantly lower compared to men [18]. Thus, the lack of mitral valve repair-related benefits including improved short- and long-term survival may be taken into account for the given results [20, 21]. Furthermore, retrospective analyses of patients treated with open heart surgery emphasize more adverse baseline conditions in women [22]. Second, regarding the overall population, patients undergoing TMVR are at prohibitive surgical risk and thus oftentimes considered inoperable. Unlike in previous analyses of patients undergoing open heart surgery, the baseline conditions in our collective were adverse in male patients [10, 18, 23]. We speculate that higher rates in comorbidities, especially of coronary artery disease and its associated deuteropathies, are accountable for wider LV dimensions, lower LV ejection fraction, and higher regurgitant volumes in men. Interestingly, similar gender-related differences were observed in the GRASP registry [13] and in a cohort recently published by Estévez-Loureiro et al. [14] without intraprocedural differences in TMVR. Patients with higher regurgitant volumes received more clips to successfully treat MR. Likewise results were seen in the EVEREST trial subanalysis [24]. Despite intraprocedural gender differences, the procedural success was high in both genders and is comparable to contemporary registers [25, 26].

Postprocedural outcomes at discharge were driven by the observation that the instantaneous effect of successful MitraClip implantation, a relative reduction of regurgitant volumes, and fractions were high in both genders (63.1% in men, 56.2% in women for regurgitant fraction and 68.6% in men, 60.3% in women for regurgitant volume). However, both parameters improved superiorly in male gender with improvement of regurgitant volume meeting significance (*p* = 0.035), likely due to initially higher baseline values and generally wider LV dimensions, and were thus driven by anatomical and not gender-specific differences. Furthermore, TSV decreased as FSV increased distinctly especially in male patients. These findings seem to be associated with a great symptomatic effect as 6MWD results of men improved whereas in the female population they almost stagnated with significantly higher values at discharge (*p* = 0.0053). A concordant effect was observed in MLHFQ with significantly lower values for men at discharge (*p* = 0.036) as previously described in general collectives [27].

Mid-term results suggest that the functional outcome of MC therapy did not reveal major gender-specific differences albeit the baseline differences of the cohorts. In analyses at 12 and 24 months, reductions in regurgitant fractions and volumes remained stable as TSV slightly and FSV greatly increased, all of which not, or only marginally, significant. This was reflected by a distinct reduction in NT-proBNP serum levels in both genders at 12 and 24 months. Again, 6MWD and MLHFQ scores developed analogically with only 6MWD showing a significantly higher value at 12 months in men (*p* < 0.001). In our opinion, the stability of initial success and the close link to symptomatic improvement reflects the benefit of MitraClip therapy even in patients at prohibitive risk rather than displaying gender-associated characteristics. The underlying cause could be attributed to improved LV hemodynamics and reverse remodeling which had previously been described following TMVR [28] even without major changes in LV ejection fractions. The positive implication of MR grade reduction on the clinical outcome confirms the findings of previously published data [29]. Stability in device success and consistent results without significant gender-related differences were also reported by Attizzani et al. [13].

Long-term analysis for survival showed a significant better outcome for female patients. One-year survival rates were slightly lower than reported in recently published data for a general collective by Swaans et al. (85.8% in the TMVR group) but substantially higher than for the conservative treatment group (67.7%) [30]. Of note, baseline parameters of the present collective in our study are eminently worse, even compared to the conservative treatment group, and more men were included. Both Kaplan-Meier curves diverge after 2 years which might be driven by the higher number of comorbidities in men as death was defined as all-cause death and was not limited to cardiac-related causes. A slight reapproximation at 4 years could support the hypothesis that medical conditions in both genders equalize in the further course and mortality is not primarily linked to the procedure. This is supported by male gender being no predictor of death in multivariate cox regression analysis. Long-term analysis for freedom from rehospitalization for heart failure did not show significant gender-related differences with minor benefits for women and again de- and reapproximation of gender-specific Kaplan-Meier curves between 3 and 4 years. These findings might contribute to the mentioned competing risks in male patients at initial presentation, thus suggesting that TMVR is beneficial in both genders in long-term outcomes. We found renal failure to be a predictor of death in both genders in univariate analysis, hinting at an association. This is of great importance, as the cardiorenal syndrome is known to interact in both directions, with failure of either organ leading to worsening of the state of the other [31]. Creatinine levels remained relatively stable in both genders throughout 24 months but were significantly higher in male patients and thus possibly explanatory for slightly worse survival rates. Atrial fibrillation, logarithmic increase of NT-proBNP serum levels, and peripheral artery disease were all gender-specific predictors of death in men. Recent findings from the ActiFE study indicated NT-proBNP to be a stronger predictor of all-cause mortality in women than in men [32]. However, as discussed, NT-proBNP is known to be associated with cardiac ischemia and atrial fibrillation [33]. Due to initially higher numbers of these comorbidities in men in comparison to women, our observation might be driven by variance. Further analyses in a larger collective are necessary.

### 4.1. Limitations

Data was obtained by a single center retrospective analysis. Therefore the study is not adequately powered to evaluate mortality and rehospitalization outcomes. Also, the given data does not represent matched patient analyses as some parameters were not obtainable due to patients missing follow-up visits or not being able to attend site visits. Furthermore, the study stretched over a period of time in which a learning curve in procedural expertise and postprocedural management occurred. Results are likely to improve in the future. In addition, our echocardiographic data was obtained in our clinical routine by experienced physicians yet was not revised by a core laboratory and thus was object to possible variance. Finally, due to low patient numbers, no subgroup analysis was performed which could lead to a more refined analysis of gender-specific data.

## 5. Conclusions

Our study demonstrates that there are no major gender-specific differences concerning (1) procedural success and (2) mid- and long-term rehospitalization for heart failure rates. However, female gender is associated with superior long-term survival. These findings should be interpreted in relation to poorer baseline conditions of the male population. The results therefore emphasize the benefit of TMVR in both genders at high surgical risk. With respect to the study limitations, the results should be regarded as hypothesis generating. Further randomized, controlled trials are necessary to refine outcome results.

## Figures and Tables

**Table 1:** Baseline patient characteristics.

	All patients (*N* = 592)	Men (*N* = 362)	Women (*N* = 230)	*p* value
Age (years)	75.0 ± 8.7	74.4 ± 8.4	76.0 ± 9.2	**0.029**
Body mass index (kg/m^2^)	25.5 ± 4.5	25.8 ± 4.1	25.0 ± 4.9	**0.031**
Logistic EuroSCORE	21.0 (11.8–33.1)	22.9 (12.2–35.9)	19.5 (11.3–29.4)	**0.020**
STS score	4.3 (2.7–7)	4.4 (2.7–7.8)	4.1 (2.7–6.1)	0.21
*Cardiovascular risk factors*				
Hypertension No. (%)	424 (71.9)	254 (70.4)	170 (74.2)	0.35
Hypercholesterolemia No. (%)	242 (41.5)	160 (44.8)	82 (36.3)	0.051
Diabetes mellitus No. (%)	164 (27.9)	119 (32.9)	45 (19.9)	**<0.001**
Nicotine abuse No. (%)	152 (41.3)	112 (51.1)	40 (26.8)	**<0.001**
*Comorbidities*				
Cardiomyopathy No. (%)	422 (73.4)	286 (80.6)	136 (61.8)	**<0.001**
Dilated	147 (25.6)	83 (23.4)	64 (29.1)	0.15
Ischemic	270 (47.0)	200 (56.3)	70 (31.8)	**<0.001**
None	153 (26.6)	69 (19.4)	84 (38.2)	**<0.001**
Coronary artery disease No. (%)	381 (64.6)	266 (73.5)	115 (50.4)	**<0.001**
Previous myocardial infarction No. (%)	195 (33.2)	145 (40.4)	50 (21.8)	**<0.001**
Previous percutaneous intervention No. (%)	238 (40.6)	166 (46.2)	72 (31.7)	**<0.001**
Previous coronary artery bypass grafting No. (%)	179 (30.3)	146 (40.3)	33 (14.4)	**<0.001**
Chronic obstructive pulmonary disease No. (%)	120 (20.4)	79 (21.9)	41 (18.0)	0.29
Renal failure No. (%)	335 (57.0)	230 (63.9)	105 (46.1)	**<0.001**
Atrial fibrillation No. (%)	398 (67.6)	250 (69.3)	148 (64.9)	0.31
Stroke No. (%)	94 (16.0)	66 (18.3)	28 (12.3)	0.068
Peripheral artery disease No. (%)	56 (9.7)	40 (11.3)	16 (7.1)	0.13
*Device*				
CRT No. (%)	119 (20.2)	86 (23.8)	33 (14.5)	**0.0091**

STS = Society of Thoracic Surgeons, CRT = cardiac resynchronization therapy, and No. = number.

**Table 2:** Intraprocedural variables.

	All patients (*N* = 592)	Men (*N* = 362)	Women (*N* = 230)	*p* value
Device time (min)	73.3 ± 52.3	77.9 ± 56.4	65.9 ± 44.2	**0.0070**
Procedural time (min)	156.3 ± 76.3	165.2 ± 78.5	142.4 ± 70.7	**<0.001**
Radiation time (min)	36.2 ± 23.0	38.6 ± 24.9	32.5 ± 19.0	**0.0024**
Number of clips (*n*)	1.4 ± 0.7	1.5 ± 0.7	1.3 ± 0.6	**0.0075**
Success No. (%)	531 (89.8)	324 (89.8)	207 (90.0)	1.00

No. = number.

**Table 3:** Postprocedural outcome, clinical variables.

	Baseline			Discharge			12 months			24 months		
	Men (*N* = 362)	Women (*N* = 230)	*p* value	Men (*N* = 362)	Women (*N* = 230)	*p* value	Men (*N* = 196)	Women (*N* = 128)	*p* value	Men (*N* = 140)	Women (*N* = 91)	*p* value
*Clinical phenotype*												
NYHA class No. (%)			0.44			0.95			0.44			0.13
I	0 (0)	0 (0)	—	4 (3.0)	3 (4.0)	1.0	17 (9.7)	12 (9.8)	1.0	18 (13.7)	6 (7.0)	0.18
II	13 (3.6)	12 (5.3)	0.44	71 (53.4)	42 (56.0)	0.83	93 (53.1)	59 (48.4)	0.49	62 (47.3)	40 (46.5)	1.0
III	243 (67.9)	160 (70.8)	0.51	54 (40.6)	28 (37.3)	0.75	56 (32.0)	48 (39.3)	0.24	41 (31.3)	37 (43.0)	0.11
IV	101 (28.2)	54 (23.9)	0.29	4 (3.0)	2 (2.7)	1.0	9 (5.1)	3 (2.5)	0.39	10 (7.6)	3 (3.5)	0.33
MLHFQ score	40.0 (27.0–52.0)	42.0 (32.0–51.0)	0.18	17.0 (15.0–19.0)	55.5 (43.8–57.6)	**0.036**	31.0 (15.0–48.0)	32.0 (11.0–47.0)	0.55	25.0 (11.0–43.5)	35.0 (24.0–44.0)	0.07
6MWD (m)	215 (79.6–320)	149 (50.0–243.8)	**0.0011**	300 (210–350)	175 (126–300)	**0.0053**	345 (180–448)	240 (150–315)	**<0.001**	355 (180–411)	270 (166–400)	0.11
*Laboratory parameters*												
NT-proBNP (ng/L)	4262 (1980–8370)	3474 (1712–6898)	0.49	4582 (2528–8609)	3512 (1896–7084)	**0.023**	2933 (1310–5062)	2331 (1324–5206)	0.79	2198 (881–4878)	2170 (1209–4277)	0.88
Troponin T (pg/mL)	35.0 (21.9–53.0)	20.0 (12.0–31.0)	**<0.001**	59.0 (39.0–89.0)	43.0 (28.0–67.3)	**<0.001**	27.0 (18.0–36.0)	17.0 (10.0–23.0)	**<0.001**	24.0 (18.4–33.0)	17.0 (12.0–26.3)	**0.0072**
Creatinine (mg/dL)	1.5 (1.2–1.9)	1.2 (0.9–1.4)	**<0.001**	1.4 (1.1–1.8)	1.0 (0.8–1.3)	**<0.001**	1.5 (1.2–1.9)	1.3 (1.0–1.6)	**<0.001**	1.5 (1.2–2.1)	1.14 (1.0–1.7)	**<0.001**
GFR (mL/min)	48.0 (36.0–62.6)	47.3 (37.3–63.2)	0.81	53.8 (39.9–70.8)	57.1 (42.2–72.7)	0.48	49.7 (36.3–61.5)	41.9 (32.3–56.6)	**0.078**	46.4 (32.1–62.3)	48.0 (32.0–57.5)	0.77
AST (U/L)	26.0 (19.0–34.8)	24.5 (18.0–33.0)	0.16	24.0 (19.0–32.0)	22.0 (17.0–28.0)	0.015	24.0 (19.0–29.0)	22.0 (18.0–27.1)	0.31	23.0 (20.0–29.0)	24.5 (19.0–28.1)	0.66
ALT (U/L)	21.0 (14.0–31.8)	19.0 (12.0–25.0)	0.018	19.0 (12.0–30.0)	16.0 (12.0–24.0)	**0.038**	19.0 (14.3–25.0)	17.0 (14.0–23.0)	0.25	22.0 (15.9–29.0)	17.5 (13.0–27.1)	0.28
gammaGT (U/L)	83.0 (45.3–160.7)	50.0 (31.0–100.3)	**<0.001**	70.5 (40.0–143.75)	49.0 (28.0–98.0)	**<0.001**	76.0 (36.2–158.0)	34.0 (23.0–68.0)	**<0.001**	58.0 (35.0–113.5)	33.5 (24.0–69.8)	**0.0020**
C-reactive protein (mg/L)	10.0 (5.0–21.0)	7.0 (5.0–13.8)	**0.014**	20.0 (12.0–43.0)	19.0 (12.0–37.2)	0.22	8.0 (5.0–13.0)	5.0 (5.0–12.1)	0.22	6.5 (5.0–13.0)	5.0 (5.0–10.3)	0.41

MLHFQ = Minnesota Living with Heart Failure Questionnaire; 6MWD = six-minute walking distance; NYHA = New York Heart Association; NT-proBNP = N-terminal probrain natriuretic peptide; GFR = glomerular filtration rate; AST = aspartate transaminase; ALT = alanine aminotransferase; and gammaGT = gamma glutamyl transferase.

**Table 4:** Postprocedural outcome, echocardiographic variables.

	Baseline			Discharge			12 months			24 months		
	Men (*N* = 362)	Women (*N* = 230)	*p* value	Men (*N* = 362)	Women (*N* = 230)	*p* value	Men (*N* = 196)	Women (*N* = 128)	*p* value	Men (*N* = 140)	Women (*N* = 91)	*p* value
MR severity No. (%)			0.16			0.21			0.77			0.21
0	0	0	—	0	0	—	1 (1)	1 (1.6)	1.00	1 (1.3)	1 (1.9)	1.00
1	0	0	—	177 (49.9)	94 (41.0)	**0.046**	31 (30.7)	15 (24.2)	0.47	26 (34.2)	12 (23.1)	0.25
2	0	0	—	140 (39.4)	107 (46.7)	0.098	60 (59.4)	37 (59.7)	1.00	43 (56.6)	29 (55.8)	1.00
3	187 (51.7)	111 (43.3)	0.47	23 (6.5)	18 (7.9)	0.64	8 (7.9)	8 (12.9)	0.44	6 (7.9)	10 (19.2)	0.10
4	175 (48.3)	117 (50.9)	0.61	8 (2.3)	3 (1.3)	0.61	1 (1)	1 (1.6)	1.00	0	0	—
Total stroke volume (mL)	69.0 (53.7–87.4)	60.2 (46.1–75.0)	**<0.001**	64.0 (54.0–78.4)	55.0 (47.2–65.8)	**<0.001**	67.1 (55.3–84.0)	65.3 (56.9–69.1)	0.20	68.8 (55.7–80.7)	57.1 (46.4–75.9)	0.11
Forward stroke volume (mL)	40.8 (31.2–52.0)	37.3 (29.5–45.4)	0.0023	52.0 (41.6–62.4)	45.4 (37.3–53.2)	**<0.001**	54.3 (43.7–66.0)	53.2 (43.7–58.9)	0.36	58.0 (48.0–68.7)	51.6 (38.0–61.2)	**0.026**
Regurgitant fraction (%)	42.5 (31.6–53.2)	38.7 (27.1–48.2)	**0.016**	18.0 (9.2–27.1)	18.0 (10.0–28.7)	0.59	19.3 (13.8–26.3)	19.4 (10.1–27.5)	0.23	16.2 (9.4–24.4)	24.1 (10.5–30.8)	0.078
Regurgitant volume (mL)	29.3 (18.7–43.9)	23.7 (13.1–36.0)	**<0.001**	11.3 (4.8–17.4)	9.6 (5.2–17.5)	0.54	13.5 (9.0–20.3)	11.7 (6.0–18.2)	0.12	10.1 (6.2–18.5)	12.7 (3.6–20.4)	0.45
Mitral valve orifice area (cm^2^)	3.8 (3.2–4.6)	3.7 (3.1–4.6)	0.90	2.9 (2.5–3.4)	2.9 (2.4–3.5)	0.77	3.0 (2.3–3.8)	2.8 (2.3–3.5)	0.69	3.0 (2.2–3.6)	2.8 (2.4–3.2)	0.35
LV end-systolic volume (mL)	113.8 (72.6–192.0)	63.4 (44.6–102.8)	**<0.001**	126.0 (78.4–201.3)	64.8 (41.8–128.1)	**<0.001**	113.3 (70.5–188.6)	66.8 (48.4–112.1)	**<0.001**	133.3 (92.2–184.3)	62.6 (48.1–115.0)	**<0.001**
LV end-systolic diameter (mm)	53.0 (45.0–63.0)	42.0 (36.0–52.6)	**<0.001**	56.0 (46.0–63.0)	46.0 (37.0–54.6)	**<0.001**	52.5 (43.0–62.6)	44.5 (35.8–54.6)	**0.0013**	54.0 (45.7–63.3)	45.0 (38.0–56.8)	**0.010**
LV end-diastolic volume (mL)	184.5 (139.4–259.9)	124.8 (95.7–169.0)	**<0.001**	190.0 (151.4–265.4)	131.2 (96.2–178.3)	**<0.001**	197.0 (145.8–245.0)	141.6 (117.5–175.8)	**<0.001**	202.0 (163.5–261.9)	140.4 (110.7–186.3)	**<0.001**
LV end-diastolic diameter (mm)	66.0 (59.0–74.0)	58.0 (51.0–64.0)	**<0.001**	66.5 (60.0–74.0)	57.5 (52.0–66.0)	**<0.001**	65.0 (57.9–72.2)	59.5 (54.0–67.0)	**0.013**	65.0 (60.0–73.6)	59.5 (54.9–67.0)	**0.0026**
LV ejection fraction (%)	39.0 ± 15.3	46.5 ± 15.1	**<0.001**	35.7 ± 14.4	44.2 ± 15.5	**<0.001**	38.9 ± 15.8	46.4 ± 15.0	**0.0092**	34.8 ± 11.8	45.6 ± 16.2	**<0.001**

LV = left ventricular; MR = mitral regurgitation.

**Table 5:** Univariate cox regression analysis for future death.

Variable	Men HR (95% CI)	*p* value	Women HR (95% CI)	*p* value
Age	1.02 (1.00–1.04)	**0.0222**	1.01 (0.99–1.04)	0.28
Body mass index	0.95 (0.91–1.00)	**0.0289 **	0.98 (0.93–1.03)	0.39
Hypertension	0.97 (0.68–1.37)	0.86	1.04 (0.63–1.71)	0.89
Hypercholesterolemia	0.62 (0.45–0.85)	**0.0031 **	1.02 (0.65–1.61)	0.94
Diabetes mellitus	1.03 (0.74–1.42)	0.87	1.78 (1.10–2.87)	**0.019 **
Nicotine abuse	1.16 (0.78–1.72)	0.46	2.04 (1.17–3.53)	**0.0115 **
Dilative cardiomyopathy	1.26 (0.88–1.78)	0.20	1.50 (0.94–2.39)	0.09
Ischemic cardiomyopathy	0.84 (0.62–1.15)	0.29	1.46 (0.92–2.34)	0.11
Coronary artery disease	0.66 (0.46–0.93)	**0.0186 **	1.40 (0.90–2.19)	0.14
Renal failure	1.79 (1.27–2.52)	**0.0009 **	1.97 (1.26–3.09)	**0.0029 **
Atrial fibrillation	1.25 (0.89–1.75)	0.19	1.41 (0.87–2.29)	0.16
MR severity 4 versus 3	1.40 (1.02–1.91)	**0.0355 **	1.23 (0.79–1.91)	0.36
LV ejection fraction < 30%	1.35 (0.97–1.90)	0.08	1.65 (0.94–2.90)	0.08
NT-proBNP (log)	1.43 (1.22–1.68)	**<0.0001 **	1.93 (1.48–2.50)	**<0.0001 **
Success rate	0.56 (0.35–0.90)	**0.0172**	0.78 (0.39–1.57)	0.49
Logistic EuroSCORE	1.00 (1.00–1.00)	0.83	1.02 (1.01–1.03)	**0.0004**
Peripheral artery disease	1.56 (0.97–2.50)	0.06	1.38 (0.67–2.88)	0.38

LV = left ventricular; MR = mitral regurgitation; NT-proBNP = N-terminal probrain natriuretic peptide; CI = confidence interval; HR = hazard ratio.

**Table 6:** Univariate cox regression analysis for future rehospitalization for heart failure.

Variable	Men HR (95% CI)	*p* value	Women HR (95% CI)	*p* value
Age	0.99 (0.97–1.01)	0.43	1.01 (0.98–1.03)	0.70
Body mass index	1.00 (0.96–1.05)	0.87	1.00 (0.95–1.06)	0.87
Hypertension	0.62 (0.41–0.94)	**0.0226 **	1.69 (0.88–3.25)	0.12
Hypercholesterolemia	1.10 (0.74–1.63)	0.65	1.60 (0.96–2.67)	0.07
Diabetes mellitus	1.07 (0.71–1.61)	0.75	1.77 (0.99–3.15)	0.053
Nicotine abuse	1.50 (0.92–2.45)	0.11	0.98 (0.44–2.15)	0.96
Dilative cardiomyopathy	1.21 (0.76–1.90)	0.42	1.68 (0.98–2.87)	0.06
Ischemic cardiomyopathy	1.07 (0.72–1.60)	0.73	1.47 (0.85–2.54)	0.17
Coronary artery disease	0.91 (0.57–1.45)	0.70	1.06 (0.64–1.78)	0.82
Renal failure	1.32 (0.88–1.98)	0.18	0.75 (0.44–1.27)	0.29
Atrial fibrillation	1.13 (0.75–1.71)	0.56	1.03 (0.61–1.75)	0.91
MR severity 4 versus 3	0.90 (0.60–1.33)	0.58	1.45 (0.87–2.43)	0.15
LV ejection fraction <30%	1.89 (1.25–2.86)	**0.0025 **	1.13 (0.56–2.25)	0.74
NT-proBNP (log)	1.29 (1.06–1.58)	**0.0115 **	1.20 (0.90–1.58)	0.21
Success rate	0.46 (0.26–0.83)	**0.0103**	0.87 (0.38–2.03)	0.76
Logistic EuroSCORE	1.00 (1.00–1.00)	0.78	1.02 (1.01–1.03)	**0.0055**
Peripheral artery disease	1.08 (0.54–2.15)	0.82	1.12 (0.45–2.80)	0.81

LV = left ventricular; MR = mitral regurgitation; MV = mitral valve; NT-proBNP = N-terminal probrain natriuretic peptide; CI = confidence interval; HR = hazard ratio.

**Table 7:** Hazard ratios for future death and rehospitalization for heart failure in men.

	Panel A		Panel B	
Variable	Stepwise backward regression analysis		^*∗*^Multivariate cox regression analysis	
	HR (95% CI)	*p* value	HR (95% CI)	*p* value
Predictors of death				
Diabetes mellitus	1.51 (1.02–2.24)	**0.037**	1.34 (0.93–1.94)	0.12
Hypercholesterolemia (%)	0.56 (0.38–0.82)	**0.0027**	0.55 (0.39–0.79)	**0.0011**
Atrial fibrillation (%)	1.64 (1.08–2.50)	**0.021**	1.46 (0.99–2.15)	0.057
NT-proBNP (log)	1.48 (1.22–1.79)	**<0.001**	1.48 (1.24–1.76)	**<0.001**
Peripheral artery disease (%)	2.16 (1.22–3.81)	**0.0078**	1.93 (1.15–3.24)	**0.012**

Predictors of rehospitalization for heart failure				
LV ejection fraction < 30%	2.46 (1.58–3.83)	**<0.0001**	1.87 (1.24–2.84)	**0.0029**
Success (%)	0.38 (0.20–0.72)	**0.003**	0.49 (0.27–0.89)	**0.019**

^*∗*^Multivariate cox regression analysis adjusted for significant predictors from stepwise backward regression analysis. CI = confidence interval.

**Table 8:** Hazard ratios for future death and rehospitalization for heart failure in women.

	Panel A		Panel B	
Variable	Stepwise backward regression analysis		^*∗*^Multivariate cox regression analysis	
	HR (95% CI)	*p* value	HR (95% CI)	*p* value
Predictors of death				
Age (years)	1.05 (1.01–1.08)	**0.0070**	1.03 (1.00–1.06)	**0.032**
Ischemic cardiomyopathy	2.26 (1.11–4.62)	**0.025**	2.25 (1.17–4.34)	**0.015**
Dilative cardiomyopathy	2.61 (1.29–5.27)	**0.0075**	2.18 (1.11–4.28)	**0.024**
NT-proBNP (log)	1.71 (1.27–2.30)	**<0.001**	1.90 (1.44–2.50)	**<0.001**

Predictors of rehospitalization				
MR severity, 4 versus 3	1.77 (0.93–3.39)	0.083	1.93 (1.04–3.60)	**0.037**
Logistic EuroSCORE	1.02 (1.00–1.03)	**0.023**	1.02 (1.00–1.03)	**0.030**
Ischemic cardiomyopathy	2.63 (1.22–5.67)	**0.013**	2.38 (1.14–4.98)	**0.021**
Dilative cardiomyopathy	3.44 (1.53–7.72)	**0.0027**	3.39 (1.57–7.30)	**0.0018**

^*∗*^Multivariate cox regression analysis adjusted for significant predictors from stepwise backward regression analysis. CI = confidence interval.

**Table 9:** Multivariate cox regression analysis for death and rehospitalization for heart failure.

Variable	Panel B	
	HR (95% CI)	*p* value
Death		
Age (years)	1.04 (1.02–1.06)	**<0.001**
Male gender	1.18 (0.82–1.68)	0.37
Body mass index	0.94 (0.90–0.98)	**0.008**
Hypertension	0.78 (0.53–1.15)	0.21
Hypercholesterolemia	0.71 (0.51–1.00)	**0.05**
Diabetes mellitus	1.56 (1.09–2.23)	**0.015**
Nicotine abuse	1.72 (1.22–2.43)	**0.0019**

Rehospitalization for heart failure		
Age (years)	1.00 (0.98–1.02)	0.87
Male gender	1.04 (0.67–1.60)	0.87
Body mass index	0.99 (0.95–1.04)	0.73
Hypertension	0.70 (0.44–1.11)	0.13
Hypercholesterolemia	1.46 (0.97–2.21)	0.07
Diabetes mellitus	1.18 (0.77–1.83)	0.45
Nicotine abuse	1.33 (0.88–2.01)	0.18

CI = confidence interval.

## Abbreviations

6MWD: Six-minute walk test
ALT: Alanine aminotransferase
AST: Aspartate transaminase
CI: Confidence interval
CRT: Cardiac resynchronization therapy
CRP: C-reactive protein
FSV: Forward stroke volume
gammaGT: Gamma glutamyl transferase
GFR: Glomerular filtration rate
HR: Hazard ratio
IQR: Interquartile range
LV: Left ventricle
M: Meter
MDRD: Modification of diet in renal disease
MLHFQ: Minnesota Living with Heart Failure Questionnaire
MR: Mitral regurgitation
NT-proBNP: N-terminal probrain natriuretic peptide
NYHA: New York Heart Association
TSV: Total stroke volume.
